# “Free Testing and PrEP without Outing Myself to Parents:” Motivation to participate in oral and injectable PrEP clinical trials among adolescent men who have sex with men

**DOI:** 10.1371/journal.pone.0200560

**Published:** 2018-07-25

**Authors:** Celia B. Fisher, Adam L. Fried, Leah Ibrahim Puri, Kathryn Macapagal, Brian Mustanski

**Affiliations:** 1 Center for Ethics Education, Fordham University, New York, New York, United States of America; 2 Clinical Psychology Department, Midwestern University, Glendale, Arizona, United States of America; 3 Feinberg School of Medicine, Northwestern University, Chicago, Illinois, United States of America; University of Toronto, CANADA

## Abstract

**Background:**

Adolescent men who have sex with men (AMSM) account for disproportionately high numbers of new HIV diagnoses. Non-adherence to daily use limiting the effectiveness of oral PrEP (Truvada) has led to current trials with adult MSM testing Cabotegravir, a long-term injectable medication. Once comparative studies with young adult MSM have established relative safety and efficacy of these medications, there will be a need for such comparative trials involving adolescents. Trends in state laws and IRB protocol review indicate that many of these studies will permit youth to provide independent consent for participation. Understanding the motivations of AMSM to participate in HIV biomedical prevention studies is important to ensure their agreement is voluntary without misunderstanding and undue influence. This study examined AMSM attitudes toward participation in oral/injectable PrEP RCTs to inform protections of youth’s rights and welfare in future studies.

**Methods:**

We administered to 198 ethnically diverse U.S. AMSM, 14–17 years, a web-based survey including demographic and sexual health questions, description of a year-long oral versus injectable PrEP RCT and 26 Likert-type and one open-ended item assessing motivations for and against participation including: perceived benefits and risks of PrEP; free HIV/STI testing and counseling; confidentiality concerns; random assignment; and benefit to others.

**Results:**

Sixty-two percent indicated they were likely to participate in the study. The majority endorsed daily HIV protection, free HIV/STI testing, sexual health counseling, not having to rely on partner’s condom use, and altruism as reasons to participate. Reasons against participation included medication side effects, concern taking the pill daily and clinic visits would reveal their sexual orientation and behaviors to parents. Over half erroneously assumed they would be assigned to the condition best for them and 39% indicated free access to services would lead them to participate even if they did not want to. Multiple regression indicated these factors accounted for 55% of the variance in participation choice. Nether age or ethnicity yielded significance.

**Conclusions:**

Results suggest future biomedical HIV prevention research will need to develop procedures to address AMSM’s confidentiality concerns, enhance youth’s understanding of random assignment, the continued importance of medication adherence and partner condom use during trial participation, and availability of alternative sexual health services to avoid the potentially undue influence of access to free sexual health services.

## Introduction

Adolescent men who have sex with men (AMSM) account for disproportionally higher numbers of new HIV diagnoses, and are more likely than their heterosexual peers to engage in high risk behaviors such as condomless sex, and to be diagnosed with a sexually transmitted infection (STI).[1–6] In addition, young MSM are less likely than older MSM to have received an HIV test and the least likely of any age group to be linked to HIV care.[6–12] The most promising U.S. Food and Drug Administration-approved biomedical prevention tool currently available is Truvada, a pre-exposure prophylaxis (PrEP) oral medication that is highly effective if taken daily.[13] However, PrEP non-adherence is prevalent among young adult MSM and uptake of PrEP has been slow due in part to required doctor visits, lack of insurance, and medical mistrust.[14–17] To address this problem, researchers are currently testing a long-term injectable PrEP medication, Cabotegravir, requiring quarterly intramuscular shots.[16–21]

In May 2018, the oral form of PrEP received FDA approval for use in adolescents.[22, 23] Along with the benefits of approval of oral PrEP for AMSM, we are likely to see adherence challenges similar to those observed in young adult populations that will surely lead to the need for oral versus injectable PrEP randomized clinical trials (RCTs) for adolescents. This research will be critical for developing effective HIV prevention programs tailored to AMSM’s developmental needs rather than simply providing services based on research with older MSM.[24–26] However, research examining the safety and efficacy of oral PrEP among AMSM has faced difficulties in recruiting youth under 18 years of age.[27–29] One recruitment barrier is the requirement for guardian permission that many AMSM fear will result in being outed to or punished by their parents.[30–34] Increasingly, states are expanding mature minor rules to include adolescent independent consent to HIV testing, treatment, and prevention.[35, 36] This will most likely lead to an increase in Institutional Review Board waivers of guardian permission and implementation of alternative protections such as participant consent advocates and assessment of youth’s independent consent capacity.

Additional protections for youth under these scenarios require understanding of ethically relevant factors that may influence their motivation to participate in PrEP medication comparative trials. Prior studies assessing adolescents’ attitudes toward participation in HIV prevention trials have focused on placebo-controlled vaccine trials or oral PrEP adherence studies.[32, 37, 38] Results of these studies suggest that the majority of youth may adequately consider personal research risks and benefits, potential future benefits to others and the nature of placebo in deciding whether to participate in HIV prevention research, but may lack understanding of randomization or hold a preventive misconception that overestimates the personal protection that is afforded by enrollment in an HIV prevention intervention trial.[37, 39, 40] Studies examining adolescent attitudes toward other types of medical research have found a majority of youth did not show evidence of therapeutic misconception [41], defined as conflating the goals of medical research and medical care.[42–44] Finally, research with adults is beginning to examine the due and undue influence of free health services on the motivation to participate in medical research, especially for populations who do not have access to health insurance.[45–48] Free health services may be a particularly motivating factor for participation of sexual minority youth who lack financial resources and health insurance to independently obtain these services. To date, however, whether any of these factors influence the decision among AMSM to participate in an RCT examining the safety and efficacy of oral and injectable PrEP has not been examined.

Understanding the motivations of AMSM to participate in HIV biomedical prevention studies is important to increasing their representation in research empirically validating prevention strategies appropriate to their developmental characteristics and life circumstances. Examining sexual minority youth’s attitudes toward biomedical HIV prevention RCTs is also necessary to ensure their agreement is voluntary without misunderstanding or undue influence. To help inform best ethical practices for future research, this exploratory study evaluated 14–17-year-old AMSMs’ motivations to participate in a hypothetical year-long RCT comparing the effectiveness and safety of oral and injectable PrEP. Prior research with adolescent and young adult MSM provided the conceptual rationale for our areas of investigation. The first area reflects studies indicating research participation is influenced by the extent to which young MSM evaluate the preventive health benefits of oral and injectable PrEP balanced by concerns regarding the continued use of condoms and medication side effects. [4, 15, 20, 34, 38] The second area draws on studies involving adults indicating that access to free health services, especially for populations like AMSM, who do not have independent access to health insurance, may be a primary motivator for research participation. [20, 45, 47–49] Our questions in this area also reflect ethics scholarship raising issues regarding the due and undue influences of such services. A third set of questions is derived from research with AMSM indicating confidentiality concerns regarding potential disclosure of sexual orientation and sexual behaviors to guardians and others as a barrier to HIV research participation. [27, 29, 30, 32, 38] Finally, we drew on the research ethics literature to develop questions examining the influence of altruism and extent to which understanding and misunderstanding of random assignment to oral and injectable PrEP conditions influence their participation decision. [37, 39, 40, 44]

## Study methods

### Participants and procedure

As part of a larger online survey study, the sample of 198 AMSM ages 14–17 years responded to paid Facebook advertisements for an Internet-based survey on attitudes toward participation in HIV prevention research and sexual health care among sexual minority youth. Participants were recruited over a 4-week period in early 2017. Interested participants completed an 11-item screening questionnaire to determine eligibility including being a cisgender male, 14–17 years old, living in the United States, being attracted to males, reporting at least one lifetime male anal sex partner, and self-reporting as HIV seronegative. Of the 1,351 individuals who clicked on the ad and completed the online screener, 959 were screened as ineligible. The majority of those screened as ineligible either did not report an anal sexual encounter with another male or were over 18; this result was not unexpected, as the ad language describing the study was intentionally broad to reduce the occurrence of desirability effects and fraudulent responding.[50, 51]

Participants who met inclusion criteria were automatically routed to the survey using LimeSurvey software. Ineligible youth were re-directed to a research participant registry with other available online studies and resources for sexual and gender minority youth. Of the 392 who completed the screener and met inclusion criteria, 156 did not complete the survey. There were no significant differences between completers and non-completers on any demographic or sexual behavior/risk variables. Of the 236 who completed the survey, 12 participants were eliminated because they failed attention and consistency validation checks, 14 were eliminated because they did not report sexual experience on the survey (would have been ineligible if reported accurately on screener) and two were not included in the analysis because they identified as female, leaving a final sample of 198.

The survey website included firewall protections with data encryption and the investigators received a Certificate of Confidentiality from the Department of Health and Human Services. Participants could end their participation at any time. All questions included the option “I do not want to answer”. Participants were provided with a $30 online gift card for their participation. The study and a waiver of guardian permission was approved by institutional review boards at Fordham University and Northwestern University Medical School.

### Demographics, family acceptance, sexual history and HIV attitudes

Participants completed questions about race, ethnicity, living situation, year in school, housing, employment and SES as measured by parents’ education. Sexual behavior and sexual health care items analyzed for this study included: (1) sexual identity, sexual history (number of lifetime sexual partners, gender of sexual partners); (2) sexual risk behaviors (anal sex without a condom and alcohol/drug use before sex); and (3) lifetime HIV testing and HIV/STI testing services received in the past year and whether they had spoken to a health practitioner about a pill called PrEP (Truvada) to prevent HIV infection. Two items assessed the extent to which youth perceived themselves at risk for HIV [52] and worried about HIV infection. Items also examined the extent to which youth were out to family members, and 5-point Likert type questions assessed their primary guardian’s acceptance of their sexual orientation and sexual activity with male partners.

### Hypothetical research vignette

The survey described different components of a PrEP RCT comparing the effectiveness of oral PrEP (taken in pill form daily) versus longer lasting injectable PrEP (injected by a physician every 3 months) to prevent acquisition of HIV. Information was presented sequentially, followed by relevant questions described below. The vignette described how youth would be randomly assigned (“like a coin toss”) to either the pill or injection condition. The study would last a year. Youth were also told the study would provide the following sexual health care services at the beginning of the study and every 3 months: HIV and STI testing, medical checkups to monitor and treat side-effects, and HIV prevention counseling on how to use condoms and other forms of protection. In addition, the vignette also explained that since PrEP is only for people who do not have HIV, youth testing positive would be excluded or withdrawn from the study and referred to a doctor for treatment. Common short-term side effects (mild headache, upset stomach, and loss of appetite) and rare side effects that could be reversed with treatment (reduced bone density, kidney health) were described. The vignette also stated that PrEP works best when other protections, such as condoms are used and that PrEP does not protect against STIs.

### Survey items

PrEP RCT items relevant to this study included 5-point Likert-type questions (1 = strongly disagree– 5 = strongly agree) reflecting motives for why youth might agree or refuse to participate in the hypothetical RCT. These items reflect fundamental principles of research ethics and [53] were developed and refined from items in prior online asynchronous focus groups and surveys on ethical issues in HIV prevention research and sexual health care involving LGBTQ youth.[32–34, 38] Six items assessed perceptions of PrEP specific health benefits and risks including: daily protection against HIV; not having to rely on a partner’s use of condoms, common and rare side effects and need to continue to use condoms for full protection against HIV and STI protection. Seven items assessed attitudes toward research that provided access to sexual health services including regularly scheduled medical checkups, sexual health counseling, being able to talk to staff affirming of youth’s sexual orientation, free HIV and STI testing, whether free access to PrEP would lead youth to participate in the study even if they otherwise would have refused and whether youth were more likely to get an HIV test from participating in the research or from their regular doctor.

Three items tapped into confidentiality concerns including fear others would find out they were participating, distrust of researchers to protect confidentiality, and concern that taking a daily pill would cause parents to start asking questions about youth’s sexual behavior. Attitudes toward random assignment to the oral pill or injection conditions were assessed through 6 items including: Whether random assignment was perceived as “fair” or would cause youth to feel like a “guinea pig”; whether knowing there was a 50% chance of assignment to the pill or injection condition would discourage participation; and youth’s belief they would be placed in the condition “best for their health needs.” Two additional items assessed motivations to participate based on altruism and logistical challenges. Two final 5-point Likert-type items (1 = definitely no– 5 = definitely yes) assessed the likelihood youth would participate in the study and whether they would still want to participate knowing that the researchers could not continue to provide PrEP services once the study was completed. Youth also responded to an open-ended question on the most important reason for their participation decision.

### Data analysis plan

Descriptive statistics were calculated for all variables. This was followed by calculating means, standard deviations and inter-item reliability for cumulative scores based on items reflecting similar motivational categories. Multivariate analysis of variance assessed whether there were demographic differences in youth’s participation choice. This was followed by Pearson product-moment correlations between the likelihood that youth would agree to participate in the hypothetical study and individual item and composite scale scores. A multiple regression was conducted to determine the extent to which cumulative scale scores and independent items together and independently accounted for variability in the likelihood of participation. The open-ended question on the most important reason for the participation decision was coded by two raters to establish inter-rater reliability of emergent themes.

## Results

### Likelihood of participation

More than half (62.6%) of AMSM indicated they would probably or definitely agree to participate in the hypothetical PrEP oral and injectable RCT; 14.6% were unsure and 22.8% would probably or definitely not participate (M = 3.64, SD = 1.23). Analysis of the open-ended question on the most important reason for the participation decision yielded good inter-rater reliability for 7 themes (Kappa range = .80–1.00). Some respondents described more than one theme. The three themes reflecting reasons to participate included protection against HIV infection, access to sexual health services, and altruism (representing 14.4%, 33.5%, and 18% of responses, respectively). Four themes reflected reasons not to participate included belief they were not at HIV risk, medication side effects and injection discomfort, confidentiality concerns and logistical concerns (8%, 4%, 16.8%, 10%, of responses, respectively). The sections below describe the relationship between the likelihood of participation and youth’s attitudes towards demographic variables, sexual behavior, attitudes toward HIV, PrEP benefits and risks, sexual health services, confidentiality concerns, random assignment, altruism and logistical challenges as well as representative quotes from the open-ended question.

### Demographics, guardian disclosure and acceptance, and sexual behavior

Frequency and percent of responses on participant demographics, guardian disclosure and acceptance and sexual history are detailed in Table 1. The majority of participants were between 16 and 17 years (M = 16.65, SD = .86), lived with a guardian/parent and identified as gay. Half the youth identified as non-Hispanic white, 34.3% as Hispanic/Latino and 14.1% as Black/African American, Asian/Pacific Islander, more than one race, or other races. The majority (74% of the 189 responding to this item) had more than one lifetime male anal sex partner and at least one anal sex experience with a male partner in which a condom was not used. Approximately half reported their guardian was both aware of and accepting of their sexual orientation. However, less than a quarter reported their guardians were aware of and accepting that they were sexually active with male partners. The majority of youth had not had an HIV test or STI test during the past year; and HIV testing during the past year was unrelated to number of unprotected anal sex experiences (*X*^*2*^_1,181_ = .13, *ns*). Neither age nor ethnicity were significantly related to other demographic, family or sexual behavior responses.

**Table 1:** Frequency and percent of responses to demographic characteristics, sexual orientation disclosure, and mean, standard deviation and range for sexual behaviors.

General Demographics	*Frequency and percent of sample (N = 198)*
**Age**	
14	8 (4.0)
15	27 (13.6)
16	80 (40.4)
17	83 (41.9)
**Race/Ethnicity**	
Black or African American	9 (4.5)
Asian/Pacific Islander	10 (5.1)
White	100 (50.5)
Hispanic/Latino	68 (34.3)
More than one race	8 (4.0)
Other	1 (0.005)
Did not respond	2 (.01)
**Living with Parents**	194 (98.0)
**Highest education of primary parent/guardian**	
High school or less	60 (30.4)
Some college	40 (20.2)
College degree	29 (14.6)
Graduate degree	64 (32.3)
**Highest education secondary parent/guardian (N = 175)**	
High school or less	55 (27.9)
Some college	22 (11.1)
College degree	36 (18.2)
Graduate degree	55 (27.8)
**Sexual Identity**	
Gay	164 (82.8)
Bisexual	26 (13.1)
Pansexual	5 (2.5)
Other	3 (1.5)
**Guardian Sexual Orientation and Activity Disclosures**	
Primary guardian definitely knows about sexual orientation	132 (66.7)
Primary guardian knows about sexual orientation and is very or somewhat accepting	107 (54.1)
Primary guardian definitely aware adolescent is sexually active with male partners	59 (29.8)
Primary guardian knows and is very is somewhat accepting of sexual activity with male partners	29 (22.7)
**Attitudes Toward HIV**	
I worry about getting infected with HIV some–all of the time	117 (59.1)
It is somewhat or extremely likely that I will become infected with HIV	29 (14.6)
**Sexual Health Services**	
Lifetime HIV Testing	69 (34.8)
Tested for HIV in past year	64 (32.3)
Tested for STI in past year	49 (24.8)
Have spoken to a health care provider about PrEP	20 (10.1)
**Sexual Health Behaviors**	
Consumed alcohol at least once before sexual contact (past year)	70 (35.4)
Consumed drugs at least once before sexual contact (past year)	50 (25.3)
Number of male anal sexual partners (lifetime)	M = 3.36
	SD = 4.58
	Range = 1–35
Number male anal sexual partners without a condom (lifetime)	M = 1.83
	SD = 2.96
	Range = 0–25

Age, race/ethnicity, living with parents, guardian education, sexual orientation and family disclosure and acceptance were not significantly associated with likelihood to participate. Only 10% of youth had discussed PrEP with a healthcare provider and this item was also not significantly associated with their participation decision. The number of lifetime male anal sex partners was positively associated with the likelihood of participation (r = .15, p < .05). Although slightly more than half the youth worried about getting infected, only 14% believed they were at risk for HIV. Worry about HIV and perceived risk of HIV infection were significantly related to the participation decision (r = .16, p < .05 and r = .21, p < .01, respectively). These 2 items were significantly correlated with one another (r = .38, p = .001; α = .55) and a composite “HIV Attitudes” score (M = 2.45, SD = .91) yielded a similar association with participation (*r* = .22, *p* < .01). Open-ended responses illustrated these attitudes: “I don’t want to take any kind of medication for things I do not have. It’s not exactly like a flu shot”, “I probably would not participate because I feel confident that I will not get HIV because I practice safe sex.” For all remaining items, means, standard deviations, percent agreement with an item and correlations with likelihood to participate are provided in Table 2.

**Table 2:** Survey item mean, standard deviation, frequency and percent agreement, and correlation with the likelihood of participating in the hypothetical study.

Variables	M (SD) ^*a*^	*N (%)* ^*b*^ *(N = 198)*	Correlation with choice
**PrEP Specific Health Benefits and Risks**			
I would have protection against HIV on a daily basis.	4.42 (0.96)	*168 (84*.*8)*	.52***
I would not have to rely on my partner using a condom to protect	3.58 (1.39)	*120 (60*.*6)*	.14**
me against getting HIV.			
PrEP works best if you also use condoms or other barrier methods,	2.18 (1.29)	*35 (17*.*7)*	-.38***
knowing this makes me less likely to be in a PrEP research study.			
PrEP alone does not protect you from other sexually transmitted.	2.12 (1.29)	*34 (17*.*2)*	-.43***
infections such as herpes or gonorrhea. Knowing this makes me			
less likely to be in a PrEP research study.			
I would worry about [brief minor] side effects (e.g. mild headache,	1.90 (1.00)	*83 (41*.*9)*	-.39***
upset stomach, loss of appetite).			
I would worry about [rare, reversible but more serious] side effects	2.35 (1.53)	*51 (25*.*7)*	-.41***
(e.g. minor decrease in bone density, minor problems with kidney			
health).			
**Access to Sexual Health Services**			
I would have a doctor check my health every 3 months.	4.09 (1.12)	*150 (75*.*8)*	.54***
I would receive sexual health counselling every 3 months.	3.71 (1.34)	*125 (63*.*2)*	.48***
I would be able to talk to research staff who are affirming of my	4.21 (0.98)	*156 (78*.*8)*	.37***
sexual orientation.			
I could get HIV testing for free.	4.42 (0.94)	*166 (83*.*8)*	.38***
I would get the PrEP medication for free.	4.33 (1.00)	*156 (78*.*8)*	.43***
Even if I did not want to be in the study, I would agree to	2.97 (1.36)	*78 (39*.*4)*	.37***
participate because it is the only way I can get PrEP for free.			
I would be more likely to get an HIV test if it was part of a PrEP	3.80 (1.15)	*141 (71*.*2)*	.31***
study than on my own.			
**Confidentiality Concerns**			
I do not trust researchers to protect my confidentiality.	2.00 (1.41)	*26 (13*.*1)*	-.43***
I'm afraid other people would find out I was participating in the	2.56 (1.41)	*70 (35*.*9)*	-.37***
study.			
If I had to take the pill every day I would worry my parents would	3.41 (1.50)	*122 (61*.*6)*	-.29***
start asking me questions about my sexual behavior.			
**Attitudes toward Random Assignment**			
I think random assignment (the coin toss) is a fair way to decide	3.48 (1.40)	*113 (57*.*1)*	.30***
who gets the injection and who gets the pill.			
Knowing I had a 50% chance of getting the pill or the injection	2.49 (1.36)	*56 (28*.*3)*	-.53***
would discourage me from participating in this PrEP study.			
I would feel the researchers were using me like a guinea pig	1.99 (1.16)	*30 (15*.*1)*	-.46***
If I was placed in the injection condition I wouldn’t want to	2.29 (1.41)	*48 (24*.*2)*	-.22**
receive a shot.			
If I was placed in the pill condition, I don't think I would.	2.50 (1.38)	*69 (34*.*9)*	-.36***
remember to take the pills everyday.			
I believe the researcher would place me in the injection or pill	3.68 (1.08)	*120 (60*.*6)*	.11
condition based upon which condition is best for my health needs.			
**Altruistic Motivation and Logistical Barriers**			
The results of the study could help other teens.	4.71 (0.58)	*185 (93*.*5)*	.36***
It would be too difficult to get to the appointments every few	2.85 (1.39)	*84 (42*.*4)*	-.49***
months.			
I do not want to know if I have HIV	1.54 (.95)	*13 (06*.*5)*	-.21**
Once the PrEP study ends, researchers cannot continue prescribing and providing PrEP, but they are able to provide a list of other doctors or clinics that can provide sexual health services, including HIV testing and PrEP, for LGBTQ teens. Knowing this, would you still want to participate in the PrEP study?	3.89 (1.16)	*140 (70*.*7)*	.82***

^a^M = Mean, SD = Standard Deviation

^*b*^*N* (%) Number and percent of AMSM responding “somewhat” or “strongly” agree with item with the following exception: for the two questions on side effects, the number and percent of AMSM responding indicates endorsement of “slightly” to “extremely” likely to worry about side effects.

*p ≥ .05

** p≥ .01

***p≥.001

### PrEP specific health benefits and risks

As indicated in Table 2, the majority of youth endorsed the value of PrEP in providing daily protection against HIV. This was illustrated in open-ended responses indicating that taking PrEP would make youth “Feel safer and more protected” and would provide “Relief of being less likely to contract HIV during sex.” Few AMSM endorsed statements indicating they would be discouraged from study participation because PrEP worked best when condoms were used and did not protect against STIs. Moreover, the majority endorsed freedom from relying on a partner to use a condom as research benefit. For some, these attitudes might lead to behavioral disinhibition as indicated by the following open-ended responses: “Not having to ruin the mood while my partner and I are having sex to make sure he put a condom on” and “I want to be safe but still able to go bareback.” One youth who indicated he was likely to participate raised an unexpected question: “If I take PrEP, do I HAVE to have sex while on the medication to participate in this study?”

Endorsement of items indicating concern about mild and more serious side effects was significantly associated with reduced likelihood of participation (Table 2). However, in their open-ended responses few AMSM mentioned side effects as a reason not to participate. Open-ended statements reflecting such concerns included: “My job, I can’t be losing any time to side effects” and “Side effects and the putting of unnatural things in my body.” A composite PrEP Specific Health Benefits score was created from the items in Table 2 listed under this category, with items related to lack of protection without a condom and side-effects reversed scored (α = .69, M = 3.91, SD = .74). The composite score was significantly associated with the likelihood of participation (*r* = .57, *p* < .001).

### Access to sexual health services

As illustrated in Table 2 the majority of respondents perceived quarterly checkups, sexual health counseling, being able to talk to research staff affirming of their sexual orientation and free access to HIV testing and PrEP medication as motivation to participate. Open-ended responses suggested youth highly valued HIV testing and sexual health counseling: “I would like to know if I have HIV and how to protect myself”, “I would like to participate so I would have a better understanding of how PrEP work[s] and how to prevent HIV”, and “Talking to health counselors about my sexual orientation.” Items reflecting free access to services were endorsed by over 80% of youth and was the most frequently mentioned reason for participating in open-ended statements as illustrated by the following: “Free resources to PrEP and prevention, free tests with someone I can learn to trust, benefit other teens, and be personally educated,” “I would get a HIV test every 3 months and I would get the medication for free.”

Almost half of AMSM would agree to participate in the study even if they did not want to because it was the only way they could get PrEP for free. As indicated in the following quote and the section below, open-ended responses suggested that for many, free access enabled them to obtain services without disclosing their sexual orientation to parents: “Free testing and PrEP without outing myself to my parents.” Almost half the youth indicated they were more likely to get an HIV test if it was part of a PrEP study than on their own. The following open-ended statement by one of the participants suggested perceived barriers to HIV preventive care might underlie these responses: “I need PrEP and my doctor won’t give it to me.” All 7 of the items in this category were positively correlated with participation choice as was the Access to Sexual Health Services composite score (α = .78; M = 3.93, SD = .75; *r* = .62, *p* < .001).

### Confidentiality concerns

As illustrated in Table 2, items reflecting confidentiality concerns were significantly associated with less likelihood of study participation. Few AMSM indicated they did not trust researchers to protect their confidentiality. However, approximately one-third were concerned that others would find out they were participating and more than half worried that taking a daily pill would lead parents to start questioning them about their sexual activity. Fear of parental disclosure was evidenced in the open-ended responses of AMSM who were unlikely to participate in the study as illustrated in following open-ended responses: “I’m very concerned about my family discovering my sexual orientation”, “The first word about LGBTQ youth and I’d most likely be disowned”, and “It goes back to confidentiality. I couldn’t participate in the study without garnering parental attention.” Confidentiality concerns were also evident in open-ended responses of AMSM who indicated they were likely to participate in the study: “Getting tested without my parents knowing”, “I want to get this free, but don’t want my parents knowing about it”, and “I would get medication for free, keep my sexual orientation from my parents, and help other people with awareness.” The Confidentiality Concerns composite score (α = .66; M = 2.65, SD = 1.05) was significantly correlated with reduced likelihood of participation (*r* = -.45 *p* < .001).

### Random assignment

As indicated in Table 2, a little more than half of AMSM believed randomization was a fair way to assign participants to groups and responses to these items were associated with increased likelihood of participating. Only 15% were concerned about being used as a guinea pig. Almost a third indicated randomization would discourage participation and endorsed items indicating reluctance to be randomized to either the pill or injection condition. These items were negatively correlated with participation. Sixty percent of the respondents believed the researcher would assign them to a condition based on what was best for their health, suggesting a conflation of research and medical care, a characteristic of therapeutic misconception. However, responses to this item were not correlated with likelihood of participating. None of the open-ended responses mentioned aspects of random assignment as a primary reason for or against participation. A Random Assignment composite score constructed from the 5 items correlated with the participation decision list in Table 2 (with the item indicating random assignment was perceived as a fair way to assign conditions, reverse scored; α = .64, M = 2.35, SD = .85) yielded a significant negative correlation with participation choice (*r* = -.57, *p* < .001).

### Altruistic motivation, testing HIV positive and logistical concerns and post-experimental access to PrEP

Almost all the youth endorsed helping other teens as a reason to participate and this item was significantly associated with likelihood of participating (see Table 2). The following are examples of open-ended responses illustrating altruistic motivation: “I would like to be a part of a community with the goal of helping other teens” and “I would like to contribute to more scientific education of LGBT teens. We don’t have much representation so this is a great study.” Close to half of AMSM endorsed an item indicating it would be too difficult to get to research required appointments and this item was significantly associated with decreased likelihood to participate. Logistical challenges were frequent among open-ended responses among youth who indicated they were unlikely to participate and often involved concern about parental disclosure or involvement as illustrated in this quote: “Getting to the check-ups without my parents finding out;” “Too hard to get to appointments, especially with my parents involved.” Although few AMSM would be reluctant to participate because they did not want to know if they had HIV, this item was significantly and negatively associated with the participation decision. A majority of youth would participate in the study knowing the researchers would provide referrals but could not continue to prescribe the PrEP once the study was over, and this item was positively associated with participation.

### Regression analyses

A multiple regression was conducted with composite scores and individual items found to be significantly correlated with the likelihood of participation were regressed onto responses to the participation item. Predictor variables included the 5 composite scales representing HIV Testing Attitudes, PrEP Specific Health Benefits, Access to Sexual Health Services, Confidentiality Concerns, Random Assignment, and single items representing number of lifetime anal sex partners, altruistic motivation and not wanting to know one’s HIV status. The post-experimental access item was not included in the regression, because unlike the other items, it was focused on on whether youth would modify their initial participation decision. The regression yielded an R^2^ = .55 (F_8,179_ = 27.44, p < .001; Durban-Watson = 1.949). As illustrated in Table 3, *Beta* scores yielding significance indicated positive independent influences on the participation choice of PrEP Specific Health Benefits and Access to Sexual Health Services and negative influence of Confidentiality Concerns.

**Table 3:** Beta coefficients for multiple regression with composite scores and single items found to be significantly correlated with the likelihood of participation regressed onto responses to the participation item.

	Unstandardized Coefficients B Std Error		Standardized Coefficients Beta	t	Sig.
(Constant)	.161	.750		0.214	.830
In your entire life, how many males (partners whose birth sex and gender identity are male) have you had anal sex with.	.009	.014	.035	.674	.501
HIV Attitudes	.079	.070	.059	1.132	.259
PrEP Health Benefits	.407	.110	.247	3.686	.000
Access Sexual Health Services	.567	.105	.347	5.404	.000
Confidentiality Concerns	-.204	.075	-.176	-2.716	.007
Random Assignment	-.195	.106	-.138	-1.831	.069
The results of the study could help other teens	.097	.123	.045	.793	.429
I do not want to know if I have HIV	-.008	.069	-.006	-.123	.902

## Discussion

HIV prevalence rates among AMSM highlight the urgent need for biomedical prevention strategies based on age appropriate and population focused research.[7] Once an injectable form of PrEP has been approved for adults, there will be a need for safety and efficacy RCTs involving at-risk AMSM comparing oral and injectable PrEP. As state laws and IRB practices continue to remove guardian permission barriers to HIV preventive interventions and research, identifying and addressing youth’s motivations for participation in PrEP biomedical trials becomes increasingly critical. To our knowledge this is the first study to examine 14–17-year-old AMSM’s motivations to participate in an oral and injectable PrEP biomedical RCT.

### PrEP specific health benefits and risks

The majority of youth endorsed daily protection against HIV. Close to half had some concern about common side effects while a quarter would worry about rare side effects, and such concerns were associated with decreased likelihood of participation. These patterns of responding suggest that AMSM are able to take into account both benefits and risks of PrEP in making informed research decisions to participate in PrEP biomedical trials. However, their responses also raise concern regarding preventive misconception and behavioral disinhibition. Prior studies focusing on attitudes of youth and young adults toward placebo-controlled HIV prevention trials have reported levels of preventive misconception (PM), defined as overestimation of personal protection in HIV prevention trials where the actual efficacy of the experimental medication is unknown.[37, 39] A strict definition of preventive misconception does not apply to our participants’ responses since the goal of the hypothetical study was to compare two existing HIV biomedical interventions found to be safe and effective for MSM only slightly older than then our respondents. However, the data point to the possibility of behavioral disinhibition in the form of reduced condom use or increased sexual activity, often associated with PM.[37, 40, 44] For example, the majority of AMSM valued PrEP as a means of not having to rely on partners use of condoms to protect them from HIV and very few considered the need to use condoms to enhance HIV/STI protections as a barrier to participation. Relatedly, in the open-ended question, one participant wrote “Do I have to have sex while I am in the study?” Such responses underscore the importance of initial consent procedures and continued counseling that provides and emphasizes the need for condom protection, respects and affirms youth’s decision to refrain from sexual activity, and includes careful monitoring throughout the study period.

### Due or undue influence of free sexual health services

Over eighty percent of AMSM endorsed receiving PrEP and HIV testing for free as a reason to participate in the PrEP prevention trial and responses to the open-ended question indicated youth associated free services with an opportunity to have HIV protection without disclosing their sexual behavior to parents. Close to forty percent indicated that free access to PrEP would lead them to participate in the study even if they did not want to. This response, within the context of youth’s endorsement of items reflecting confidentiality concerns, raise questions about whether or not free services exert an undue influence over participants whose age, sexual minority and insurance status may make them particularly vulnerable to exploitation.[54]

There are varying definitions of undue influence in research, most of which have focused on the use of monetary incentives.[55, 56] To date, little attention has been paid to whether the opportunity to participate in trials offering free biomedical HIV prevention and referral services exerts an undue influence on sexual minority adolescents who lack financial resources and health insurance necessary to independently obtain these services. Most relevant to the issues raised by the hypothetical PrEP RCT is whether the offer to provide free HIV prevention services results in an agreement to participate based on an unreasonable devaluing of potential research risks, or whether the explanation of risks and benefits within the context of the availability of such services leads youth who might have originally considered declining to make a reasonable adjustment to their risk-benefit analysis.[46, 57] Potential remedies include implementing strategies that ensure that risks are well-understood by potential participants during consent procedures and that monitor and address throughout the course of the study youth’s reactions to medication side effects as well as potential negative family or social reactions to youth’s participation.

At tension with concerns about undue influence is providing AMSM fair access to research critical to future effective prevention strategies.[24, 27] A careful analysis of the nature of the hypothetical randomized trial indicates it meets criteria for fair selection and participant protection.[47, 58] First, AMSM will be recruited for such studies, not as a sample of convenience, but because their sexual behaviors and vulnerability to family and social stigma place them at increased HIV risk and require evidence-based, age appropriate prevention strategies tailored to their needs. Second, since the safety and efficacy of medications will have been documented for young adults, the study has a favorable benefit-risk ratio that would apply to both those with and without the ability to independently obtain sexual health services.[49] Third, although some have argued that lack of affordable post-trial access can lead to exploitation [56] to date in most states these products are currently unavailable to youth, irrespective of their insurance status. This does not address the financial health inequities that AMSM may continue to face once the medications have been approved.[49, 59] It does require investigators to ensure that participants are able to make a risk-benefit analysis specific to their personal needs, to know there is no guarantee of post-experimental services, and to work with local health care institutions to be able to provide referrals to public assistance or free clinics if available.[47]

### Confidentiality concerns

Our data is consistent with previous work indicating that confidentiality concerns about disclosure of sexual orientation and sexual behaviors to guardians and fear of stigma associated with HIV risk present significant barriers to AMSM’s participation in HIV prevention research.[27, 29, 30, 32, 33, 38] Over half the participants expressed concern that taking the PrEP pill daily might inadvertently risk disclosure of sexual behaviors to parents. An even greater number of youth indicated that getting to required clinic visits without risking disclosure to guardians would prevent participation. These disclosure fears have implications not only for research participation, but for AMSM’s future use of oral and injectable PrEP prescribed by physicians. These findings suggest that research staff should help youth develop pill adherence strategies that do not risk disclosure as well as consider providing transportation for youth to clinics or otherwise making clinic visits easily accessible. If effective, such procedures have the potential to be adapted by sexual health care providers.

### Random assignment and therapeutic misconception

Responses suggest youth appreciated the implications of random assignment and were able to make a reasoned decision to refuse participation if they did not want to be assigned to the pill or to the injection condition.[52] However, over half assumed the investigator would assign them to the condition best for their individual health needs. Although responses to this last item were not significantly correlated with their participation choice, it raises questions regarding therapeutic misconception.[42, 60] Even when youth benefit and suffer little or no physical harm from participation, they may be wronged if they do not understand that research is not the same as treatment.[43] The apparent contradiction between youth’s understanding of randomization and misperception of personal medical attention may be a function of the fact that while the hypothetical study adequately described the nature of the two experimental conditions and provided the coin toss analogy, it did not specifically address the difference between research and medical treatment. This suggests that informed consent for future studies move beyond the “coin toss” explanations for random assignment and include discussion and opportunities for questions regarding the distinction between research and personally tailored medical services.

### Strengths and limitations

Our online data collection and recruitment methods yielded a national sample of sexually active AMSM; however, this methodology does not allow for absolute certainty that inclusion criteria were met and also limited participation to those with Internet or mobile phone access, and those who frequent social media. Second, although half our sample self-identified as racial/ethnic minorities, there were insufficient respondents from specific ethnic groups to examine issues related to how the intersections of ethnic and sexual minority status may influence motivation to participate in HIV research.[10, 25] Third, almost all youth lived with their parents, the majority of whom had at least some college education. Thus, our study may not have captured the views of low-income or homeless youth, youth who have been abandoned by their families, or who may be engaged in sex work or other sexual behaviors and would most benefit from prevention strategies tailored to their lived experience. Fourth, our study did not focus on an assessment of AMSM’s understanding of the different research components of the hypothetical RCT. Previous research applying quantitative and qualitative methods to explore youth’s ability to provide informed consent to HIV vaccine and oral PrEP adherence studies, suggest they are able to understand research risks and benefits of such research while at the same time, consistent with our data, may be vulnerable to reduced use of condoms.[32, 37] Future research employing qualitative methods will provide greater depth knowledge of the extent and limits of youth’s understanding of PrEP pill and injectable comparative studies. Fifth, 0ur study did not assess the impact of state mandatory disclosure requirements involving reporting of positive HIV or STI status or child abuse on the participation choice. Confidentiality concerns in response to informed consent describing these mandates may increase AMSM’s reluctance to participate in HIV prevention research. Finally, once comparative PrEP studies are approved, it will be important to assess the generalizability of youth’s response to the hypothetical scenario to their actual participation decisions.

## Conclusion

The urgent need to address the HIV epidemic among sexual minority adolescents in the United States requires empirically validated biomedical prevention strategies based on the youth’s unique characteristics and life circumstances.[24, 27] Once funding for comparative studies on the safety and efficacy of oral and injectable PrEP is approved for adults, understanding the motivations of AMSM to participate in these trials will be essential to ensure youth’s participation is informed and voluntary. The promise of PrEP trials in providing empirically based preventive interventions for high risk youth should not be overshadowed by the importance of designing developmentally appropriate ethical procedures.[61] Taken together our data suggest that youth’s participation decisions for PrEP biomedical studies will reflect an understanding and appreciation of the benefits and risks of PrEP medications, the value of research that provides HIV testing and sexual health services, and a desire to help other sexual minority youth. The reasonableness of these motives to participate is, however, tempered by the potential for confusing random assignment with personal care, decrease in condom use during sex with male partners, and the potential undue influence of free sexual health services.

Threats to informed and safe participation posed by therapeutic misconception, and the potential for behavioral disinhibition can be adequately addressed through developmentally and sexual orientation appropriate sexual health literacy enhancement procedures during recruitment and consent, and continued monitoring procedures.[38, 62] Although investigators may be able to mitigate the influence of free services on participation decisions through informed consent policies alerting youth to public assistance programs or free clinics, these efforts will remain inadequate as long as there continues to be financial disparities, fear of parental disclosure and other systemic barriers to sexual health services affecting the lives of AMSM.[47, 49, 59] It is important to note that potentially problematic motivations such as therapeutic misconception, behavioral disinhibition, and access to free services have been well documented in adult populations [44, 45, 48, 60, 63] and are thus not a rationale for over-estimating the vulnerability of adolescents and thereby excluding them from participation in studies essential to the health of AMSM in the future. On the contrary, our data suggest that randomized comparative biomedical HIV prevention trials tailored to the developmental needs of sexual minority youth may not only be important for establishing appropriate evidence-based services but also serve as a critical gateway for HIV testing, prevention services and counseling, and when appropriate, HIV treatment referrals for this underserved population.

## Supporting information

S1 File Questionnaire. (DOCX)

S2 File Data included subjects. (SAV)

S3 File Data excluded subjects. (SAV)
