# Supplementary material for: “Free Testing and PrEP without Outing Myself to Parents:” Motivation to participate in oral and injectable PrEP clinical trials among adolescent men who have sex with men
Source: PLoS One. 2018 Jul 25;13(7):e0200560. doi: 10.1371/journal.pone.0200560 (PMC6059443; doi:10.1371/journal.pone.0200560)
Supplement: S1 File — (DOCX) [file pone.0200560.s001.docx]

**The Adolescent Scientific Access Project (ASAP!)**

**Ethics in HIV Prevention Research Involving LGBTQ Youth**

Northwestern University IRB# STU94979

Principal Investigators: Dr. Brian Mustanski (Northwestern University)

Dr. Celia B. Fisher (Fordham University)

Your opinions are needed! We want to know how young lesbian, gay, bisexual, transgender, or queer (LGBTQ) people feel about being in sexual health and HIV prevention research. Your opinions can help LGBTQ youth like you take part in research studies that can improve their health.

**How does it work?** 
If you qualify for the study and agree to take part,

- You’ll do a 25-30 minute online survey. The survey asks about things related to being LGBTQ, your health and sexual behavior, and what you think and feel about studies related to HIV prevention.
- If you complete the entire questionnaire, you can receive a $30 gift card.
- If you’re completing this on a mobile device or smartphone, please click “Next” at the bottom of each page to go onto the next question.

Please note: You do not qualify for this study if you previously participated in an online survey for LGBTQ teens from the Adolescent Scientific Access Project (ASAP) at Northwestern University.

**PTP1.** Would you like to complete this form to find out if you are eligible to participate?

- **(1)** Yes
- **(0)** No *ineligible*

***(PTP1 = 1)*** Thanks for your interest in being involved in the ASAP study. We are now going to ask you a few questions to see if you are eligible to be in the study.

1. **RA1** Please pick the word that best fits what is shown in the corresponding photo. ***[RANDOMIZED OPTIONS]***

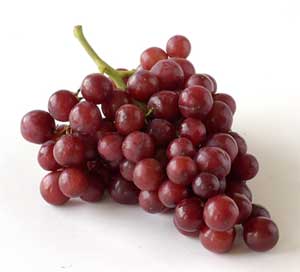


- **(1)** Cherries *(ineligible)*
- **(2)** Running *(ineligible)*
- **(3)** Grapes
- **(4)** Archery *(ineligible)*

1. **RA2** Please pick the word that best fits what is shown in the corresponding photo. ***[RANDOMIZED OPTIONS]***

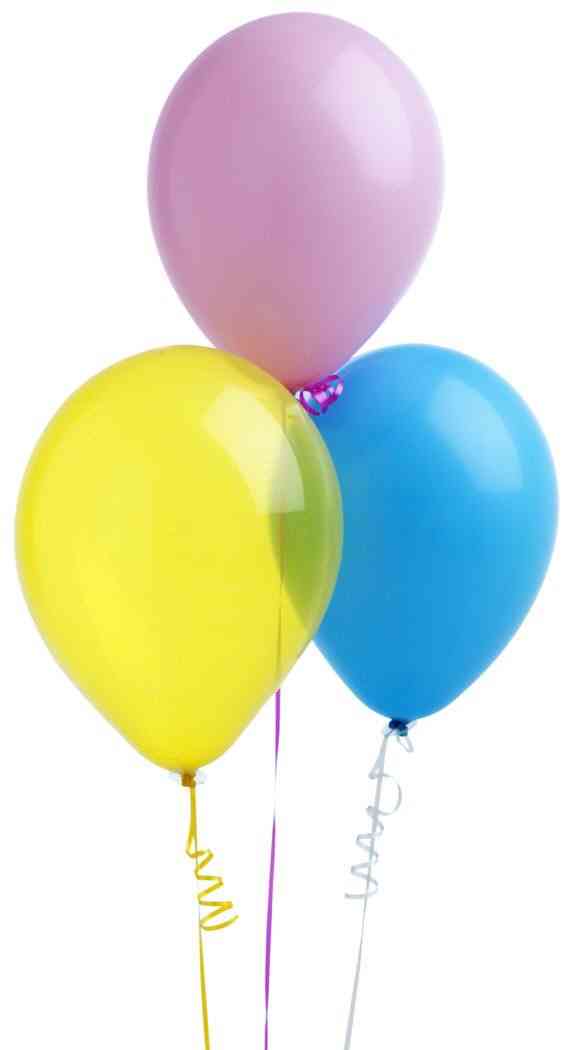


- **(1)** Flying kite *(ineligible)*
- **(2)** Spinning top *(ineligible)*
- **(3)** Several balloons
- **(4)** An old drum *(ineligible)*

1. **RA3** Please pick the word that best fits what is shown in the corresponding photo. ***[RANDOMIZED OPTIONS]***

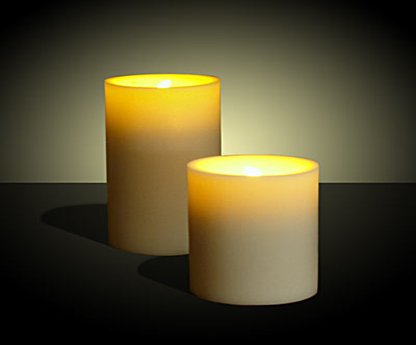


- **(1)** Trash basket *(ineligible)*
- **(2)** Silver spoon *(ineligible)*
- **(3)** Crystal glass *(ineligible)*
- **(4)** White candles

**Now, we would like to get some general information about you to determine whether you are eligible for this study. Please answer all questions as best as you can. Your responses are confidential and used only for the purposes of this study. If you are not eligible for this study, we will not keep any of your information.**

1. **Demo3Age** How old are you?

*(<14 or > 17 = ineligible)*

1. **Demo5** Are you of Hispanic or Latino ethnicity?
   - **(0)** No
   - **(1)** Yes
   - **(99)** *I do not want to answer*
2. **Demo4** What is your race?
   - **(1)** White
   - **(2)** Black or African American
   - **(3)** Asian
   - **(4)** Native Hawaiian or Other Pacific Islander
   - **(5)** American Indian/Alaska Native
   - **(6)** More than one race
   - **(7)** Other
   - **(99)** *I do not want to answer*
3. **Demo4mult.** You selected "more than one race." Please specify:

*If you do not wish to answer this question, type "no answer" in the text box.*

1. **Demo4oth.** You selected "other." Please specify the term you use to describe your race.

*If you do not wish to answer this question, type "no answer" in the text box.*

1. **Demo6** Which of the following best describes your living situation?
   - **(1)** Living alone in an apartment, dorm, or house
   - **(2)** Living with parents or other family members
   - **(3)** Living with a roommate in an apartment, dorm, or house
   - **(4)** Living with a romantic or sexual partner
   - **(5)** Living in a shelter, group home or residential treatment facility *(ineligible)*
   - **(6)** No permanent address (homeless, squatting, etc.) *(ineligible)*
   - **(99)** *I do not want to answer* *(ineligible)*

**The sex assigned to you on your birth certificate may be the same or different from your gender identity.**

1. **Demo7** What was the sex assigned to you on your birth certificate?
   - **(1)** Male
   - **(2)** Female *(ineligible)*
   - **(3)** Other *(ineligible)*
   - **(99)** *I do not want to answer* *(ineligible)*

**Gender identity refers to cultural values (roles, behaviors, activities, and attributes) that a society associates with being male or female. Your gender is how you feel inside and can be the same or different than the answer you gave above.**

1. **Demo8** For research purposes and to better understand the individuals completing our survey, please describe your gender identity using these commonly used categories.
   - **(1)** Man
   - **(2)** Woman *(ineligible)*
   - **(3)** Transgender Man (female to male; FTM) *(ineligible)*
   - **(4)** Transgender Woman (male to female; MTF)
   - **(5)** Genderqueer *(ineligible)*
   - **(6)** Gender nonconforming *(ineligible)*
   - **(7)** Not listed *(ineligible)*
   - **(99)** *I do not want to answer* *(ineligible)*
2. **Demo23** Have you ever had sexual re-assignment surgery?
   - **(0)** No
   - **(1)** Yes *(ineligible)*
   - **(99)** *I do not want to answer* *(ineligible)*
3. **Demo10** Which of these commonly used labels best describes your sexual orientation?
   *Please note that you will have another opportunity to share other labels you use to describe your sexual orientation in the following question.*
   - **(1)** Gay
   - **(2)** Lesbian *(ineligible)*
   - **(3)** Bisexual
   - **(4)** Pansexual
   - **(5)** Asexual
   - **(6)** Heterosexual (straight)
   - **(7)** Queer
   - **(8)** Questioning/Unsure
   - **(99)** *I do not want to answer* *(ineligible)*
4. **Demo11** If there are other terms besides the ones above that you use to describe your sexual orientation, please specify those here:

*If you do not wish to answer this question, type "no answer" in the text box.*

***We have just a few more questions to see if you are eligible for the study.***

1. **Demo12** Who have you had vaginal or anal sex with in your lifetime?
   - **(1)** Only guys
   - **(2)** Mostly guys, but some girls
   - **(3)** Guys and girls equally
   - **(4)** Mostly girls, but some guys
   - **(5)** Only girls *(ineligible)*
   - **(88***Not applicable, I have never had sex* *(ineligible)*
   - **(]….**  *I do not want to answer* *(ineligible)*
2. **Demo14** What was the result of your most recent HIV test? Remember, your answers are confidential.
   - **(1)** Negative
   - **(2)** Positive *(ineligible)*
   - **(3)** I don’t know
   - **(8** *Not applicable; I have not been tested for HIV*
   - **…..** *I do not want to answer* *(ineligible)*
3. **SOD01** How out are you to people around you about your sexual orientation?
   - **(1)** Not out to anyone
   - **(2)** Only out to a select few people
   - **(3)** Out to most people
   - **(4)** Out to everyone
   - **(99)** *I do not want to answer*

***Don't give up now! Last page before you find out if you are eligible!***

**If our security measures detect that you have previously participated in this study you will not be eligible to receive payment.**

1. **RES3** Have you ever been in a research study with the **IMPACT Program at Northwestern University** where you were asked to participate in 1) an online focus group with other LGBTQ teenagers and/or 2) an online survey about sexual behavior and HIV in LGBTQ teenagers?
   - **(1)** Yes *(ineligible)*
   - **(0)** No
   - **..**  *I do not want to answer (ineligible)*
2. **RES4** Have you ever been in a research study with the **Fordham University** Center for Ethics Education where you filled out a health survey for transgender and gender nonconforming youth?
   - **(1)** Yes *(ineligible)*
   - **(0)** No
   - **(99)** *I do not want to answer (ineligible)***ens ineligible, participants see this**

Thank you for your time! Unfortunately, your responses indicate that you are not eligible to participate in this particular study. But, we often are looking for participants for our other studies.

**EN**

**Congratulations!**

**Your responses suggest that you qualify for our study!**

http://giphy.com/gifs/happy-excited-spongebob-squarepants-8WJw9kAG3wonu

The rest of the survey will take about 25-30 minutes. You may save your progress and return to your spot later by clicking the "Resume Later" button in the upper right hand corner. If you do so, you will be given a password and asked to set a username; you'll also have the option of having this information emailed to you. **Be sure to save your username and password so that you can log in later**!

On the next page, you will see a consent form. This form tells you more details about the study, its risks and benefits, your rights as a participant, and what to do if you have questions or concerns. If you agree to participate after reading this form, you will be taken straight to the survey.

**Click next at the bottom of the page to view the consent form.**

**[CONSENT FORM]**

**Consent.**

- If you want a copy of this consent for your records, you can print it from the screen.
- If you wish to participate, please click the “I Agree” button and you will be taken to the survey.
- If you do not wish to participate in this study, please select “I Disagree” or select X in the corner of your browser.
  - **(1)** I Agree
  - **(0)** I Disagree *(ineligible, skip to END)*

***(Consent = 0)* consentno03.** Thanks for your time!

***(Consent = 0) – END of survey for those who said no. They now see the message saying their responses have been recorded,***

***(Consent= 1)***

**We are interested in how your relationship with your parents might be related to your thoughts and feelings about sexual health and HIV research. Your answers to the following questions can help us better understand who your parents are and their role in your life.**

Type the first name of up to **2** people you would consider your parents, legal guardians, or primary care takers. If you do not wish to type in their names, you can write in a nickname you use to refer to them.

Legal guardians are the main adults who are legally responsible for you. They do things like sign permission slips for school. They also can take care of you in an emergency. Your legal guardian(s) can be someone who is related to you, or someone else.

**--- PAGE BREAK ---**

1. **GuardianDem01b**  Of the options below, please select one term that describes **{GuardianDem01a’s}** role:

- **(1)** Mom
- **(2)** Dad
- **(3)** Parent
- **(4)** Stepmom
- **(5)** Stepdad
- **(6)** Stepparent
- **(7)** Foster Parent
- **(8)** Co-parent or Other parent
- **(9)** Other family member (grandparent,
   aunt/uncle, sibling, cousin)
- **(10)** Family Friend
- **(11)** Other person not listed
- **(99)** *I do not want to answer*

1. **GuardianDem01d** What is the highest level of education that **{GuardianDem01a}** has completed?

- **(1)** 8th grade or less
- **(2)** Partial high school
- **(3)** High school graduate
- **(4)** Partial college (at least one year)
- **(5)** Undergraduate college degree
- **(6)** Graduate degree
- **(7)** I don’t know
- **(99)** *I do not want to answer*

1. **GuardianDem02b** Of the options below, please select one term that describes **{GuardianDem02a’s}** role:

- **(1)** Mom
- **(2)** Dad
- **(3)** Parent
- **(4)** Stepmom
- **(5)** Stepdad
- **(6)** Stepparent
- **(7)** Foster Parent
- **(8)** Co-parent or Other parent
- **(9)** Other family member (grandparent,
   aunt/uncle, sibling, cousin)
- **(10)** Family Friend
- **(11)** Other person not listed
- **(99)** *I do not want to answer*

1. **GuardianDem02d** What is the highest level of education that **{GuardianDemo02a}** has completed?

- **(1)** 8th grade or less
- **(2)** Partial high school
- **(3)** High school graduate
- **(4)** Partial college (at least one year)
- **(5)** Undergraduate college degree
- **(6)** Graduate degree
- **(7)** I don’t know
- **(99)** *I do not want to answer*

**To help us understand factors that may influence your participation in sexual health and HIV prevention care and research, we need to ask about your parents’ knowledge of your sexual activity in general and about your sexual orientation.**

1. **SOD13** Does **{GuardianDem01a}** know that you are sexually active with male partners?

- **(1)** Definitely knows and we have talked about it
- **(2)** Definitely knows and we have never talked about it
- **(3)** Probably knows or suspects
- **(4)** Does not know or suspect
- **(99)** *I do not want to answer*

**--- PAGE BREAK**

1. **SOD14** How accepting is **{GuardianDem01a}** that you are sexually active with male partners?
   - **(1)** Very rejecting
   - **(2)** Somewhat rejecting
   - **(3)** Neither rejecting nor accepting
   - **(4)** Somewhat accepting
   - **(5)** Very accepting
   - **(99)** *I do not want to answer*
2. **SOD02** Does **{GuardianDem01a}** know about your sexual orientation?

- **(1)** Definitely knows and we have talked about it
- **(2)** Definitely knows and we have never talked about it
- **(3)** Probably knows or suspects
- **(4)** Does not know or suspect
- **(99)** *I do not want to answer*

**--- PAGE BREAK -**

1. ***(*SOD04** How accepting is **{GuardianDem01a}** of your sexual orientation?
   - **(1)** Very rejecting
   - **(2)** Somewhat rejecting
   - **(3)** Neither rejecting nor accepting
   - **(4)** Somewhat accepting
   - **(5)** Very accepting
   - **(99)** *I do not want to answer*
2. ***(*SOD16** Does **{GuardianDem02a}** know that you are sexually active?

- **(1)** Definitely knows and we have talked about it
- **(2)** Definitely knows and we have never talked about it
- **(3)** Probably knows or suspects
- **(4)** Does not know or suspect
- **(99)** *I do not want to answer*

**The following questions ask about your experiences as an LGBTQ person receiving services from medical health care providers. A health care provider is a medical doctor, nurse practitioner, or other person providing medical care.**

1. **HC02d** In the past I have spoken to a doctor, nurse or other healthcare provider about taking a pill called PrEP (e.g. Truvada) to prevent HIV infection.

- **(1)** Never
- **(2)** Rarely
- **(3)** Sometimes
- **(4)** Often
- **(5)** Always
- **(99)** *I do not want to answer*

1. **ATTN01** When responding to this next question, please pick the response option that says "Rarely" so that we know you are reading these questions.
   - **(1)** Never
   - **(2)** Rarely
   - **(3)** Sometimes
   - **(4)** Often
   - **(5)** Always
2. **HC07** Have you been tested for HIV in your life?
   - **(1)** Yes
   - **(0)** No
   - **(2)** I don’t know
   - **(99)** *I do not want to answer*

**-- PAGE BREAK --**

1. ***(*HC08** Have you been tested for HIV in the past year?
   - **(1)** Yes
   - **(0)** No
   - **(2)** I don’t know
   - **(99)** *I do not want to answer*

**--- PAGE BREAK ---**

1. **HC09** In the past year have you been tested for another sexually transmitted infection (STI) such as gonorrhea, chlamydia, syphilis, trichomonas, genital herpes, genital warts, or others?
   - **(1)** Never
   - **(2)** Rarely
   - **(3)** Sometimes
   - **(4)** Often
   - **(5)** Always
   - **(9**  *I do not want to answer*

**--- PAGE BREAK ---**

1. **HC11** How likely do you think you are to become infected with HIV?
   - **(1)** Extremely unlikely
   - **(2)** Somewhat unlikely
   - **(3)** Neither likely nor unlikely
   - **(4)** Somewhat likely
   - **(5)** Extremely likely
   - **(99)** *I do not want to answer*
2. **HC12** How much do you worry about getting infected with HIV?
   - **(1)** None of the time
   - **(2)** Rarely
   - **(3)** Some of the time
   - **(4)** A lot of the time
   - **(5)** All of the time
   - **(99)** *I do not want to answer*

**To help us better understand the responses provided by the people who fill out this survey, we need to ask questions about sexual behaviors that can lead to sexual health risks, like HIV or other sexually transmitted infections.**

**HIV and other STIs are typically transmitted through sexual activity that includes contact with or exchange of genital fluids such as semen and vaginal fluid. Such sexual contact includes penile-vaginal sex (when someone puts their penis in someone else’s vagina) and anal sex (when someone puts their penis in someone else’s butt). Please answer the following questions as honestly and accurately as you can.**

1. **SEX2a** In your entire life, how many males (partners whose birth sex and gender identity are male) have you had anal sex with?

*If the answer is "none" please do* ***not*** *leave blank. Instead, enter "0"*

1. **SEX4a** Of the **{Sex2a}** **males** you had anal sex with, with how many did you have anal sex without a condom or other protective barrier?

*If the answer is "none" please do* ***not*** *leave blank. Instead, enter "0"*

1. **SUB1** In the PAST 12 MONTHS, how frequently did you drink alcohol before having sexual contact with a male or transgender female partner?

- **(1)** Never
- **(2)** Rarely
- **(3)** Sometimes
- **(4)** Often
- **(5)** Always
- **(99)** *I do not want to answer*

1. **SUB2** In the PAST 12 MONTHS, how frequently did you use drugs before having sexual contact with a male or transgender female partner?

- **(1)** Never
- **(2)** Rarely
- **(3)** Sometimes
- **(4)** Often
- **(5)** Always
- **(99)** *I do not want to answer*

1. **ATTN02** Even if these instructions seem strange to you, please pick the response option that says "strongly agree" below.
   - **(1)** Strongly disagree
   - **(2)** Somewhat disagree
   - **(3)** Neither agree nor disagree
   - **(4)** Somewhat agree
   - **(5)** Strongly agree

**Now, we would like to hear your opinions about participating in an HIV prevention research study for LGBTQ teens. Just being LGBTQ does not automatically put you at risk for HIV. HIV can affect anyone who is sexually active, particularly when people have sexual contact without protection such as condoms. It is important to know that people with HIV can live happy and healthy lives taking HIV medications.**

**There is a medication called PrEP (pre-exposure prophylaxis) that can help protect sexually active teens from becoming infected with HIV. PrEP can be given in pill form every day or by a doctor giving you an injection every 3 months. PrEP works best if it is taken used with other types of protection like condoms.**

**PrEP does not protect you from other sexually transmitted infections (STIs) like herpes or gonorrhea. So anyone who is sexually active should always use condoms or other barrier methods.**

1. **PrEP3** PrEP works best if you also use condoms or other barrier methods.
   Knowing this makes me less likely to be in a PrEP research study.

- **(1)** Strongly disagree
- **(2)** Somewhat disagree
- **(3)** Neither agree nor disagree
- **(4)** Somewhat agree
- **(5)** Strongly agree
- **(99)** *I do not want to answer*

1. **PrEP4** PrEP alone does not protect you from other sexually transmitted infections such as herpes or gonorrhea.
   Knowing this makes me less likely to be in a PrEP research study

- **(1)** Strongly disagree
- **(2)** Somewhat disagree
- **(3)** Neither agree nor disagree
- **(4)** Somewhat agree
- **(5)** Strongly agree
- **(99)** *I do not want to answer*

**The PrEP Study:**

**Researchers are interested in assessing how well PrEP can reduce risk of HIV transmission among LGBTQ teens. Teens who agreed to participate in this study would do the following things.**

- **Since PrEP only works for people who do not have HIV, teens would need to have an HIV test before they can participate.**
- **If the test confirms the teen does not have HIV, they can join the study and begin taking PrEP.**
- **Teens would be in the PrEP study for 1 year.**
- **Teens would get tested for HIV every 3 months that they are on PrEP.**
- **If the tests show that the teen has HIV, the researchers will connect them with a doctor who can give them the right treatment for HIV.**

**When answering the questions below, imagine if you were asked to be in a PrEP study for LGBTQ youth.**

1. **PREP8** I would be more likely to get an HIV test if it was part of a PrEP study than on my own.
   - **(1)** Strongly disagree
   - **(2)** Somewhat disagree
   - **(3)** Neither agree nor disagree
   - **(4)** Somewhat agree
   - **(5)** Strongly agree
   - **(99)9***I do not want to answer*

**PrEP protects against getting HIV, but it does NOT protect against other STIs or pregnancy. Everyone who is in a PrEP study also gets counseling at every check up on how to use condoms and other forms of protection.**

**Most people who take PrEP don't experience side effects. Some people on PrEP experience short term side effects like mild headache, upset stomach, and loss of appetite that go away a few weeks after beginning the medication.**

1. **PREP12** I would worry about the side effects above.
   - **(0)** Not at all
   - **(1)** Slightly
   - **(2)** Moderately
   - **(3)** Considerably
   - **(4)** Extremely
   - **(99)9***I do not want to answer*

**Some people also experience a minor decrease in bone density, though this has not been found to be a serious cause of concern. Around 1 in 200 people have minor problems with kidney health, which got better when they stopped PrEP. Every 3 months teens participating in the PrEP study would receive a medical checkup by the research team to monitor and treat any side effects.**

1. **PREP13** I would worry about the side effects above.
   - **(0)** Not at all
   - **(1)** Slightly
   - **(2)** Moderately
   - **(3)** Considerably
   - **(4)** Extremely
   - **(99)9***I do not want to answer*

**One type of study that is currently being conducted tests whether PrEP pills or injections are better in reducing HIV risk among sexually active teens whose partners are male.**

**The PrEP pills must be taken every day, while the injections are given to teens by a doctor once every 3 months. To know which method works best, half (50%) of the teens would get the injection and half (50%) would receive the pills.**

**Which teens received the injection and which received the pills would be selected at random, like a coin toss.**

- **Teens in both conditions would come to the study site every 3 months to get tested for HIV and to receive a medical check-up and sexual health counseling.**
- **At each visit teens in the PrEP pill condition would receive a 3 month prescription for the pills and teens in the PrEP injection condition would receive the injection by a doctor on the research team.**
- **The potential side effects for both methods are the same (mild headache, upset stomach, loss of appetite, and in rare cases minor decrease in bone density and kidney problems.**
- **At each visit, doctors would test for and treat any negative side effects. For teens receiving the injection, there may also be short-term pain at the injection site.**

1. **PREP15** Knowing I had a 50% chance of getting the pill or the injection would discourage me from participating in this PrEP study.
   - **(1)** Strongly disagree
   - **(2)** Somewhat disagree
   - **(3)** Neither agree nor disagree
   - **(4)** Somewhat agree
   - **(5)** Strongly agree
   - **(99)9***I do not want to answer*
2. **PREP16** I think random assignment (the coin toss) is a fair way to decide who gets the injection and who gets the pill.

- **(1)** Strongly disagree
- **(2)** Somewhat disagree
- **(3)** Neither agree nor disagree
- **(4)** Somewhat agree
- **(5)** Strongly agree
- **(99)** *I do not want to answer*

**For teens in the pill condition, the PrEP pill must be taken every day to protect against HIV**

1. **PREP11** If I had to take the PrEP pill every day, I would worry my parents/guardians would start asking me questions about my sexual behavior.

- **(1)** Strongly disagree
- **(2)** Somewhat disagree
- **(3)** Neither agree nor disagree
- **(4)** Somewhat agree
- **(5)** Strongly agree
- **(99)** *I do not want to answer*

1. **PREP38** I believe the researcher would place me in the injection or pill condition based upon which condition is best for my health needs.

- **(1)** Strongly disagree
- **(2)** Somewhat disagree
- **(3)** Neither agree nor disagree
- **(4)** Somewhat agree
- **(5)** Strongly agree
- **(99)** *I do not want to answer*

**The following are reasons you might WANT to participate in this type of study that tests whether PrEP pills or injections are the best way to protect teens who have sex with male partners from getting HIV. Please indicate how much you agree with these reasons.**

**A benefit of participating in this study is...**

1. **PREP17b.** I could get HIV testing for free
   - **(1)** Strongly disagree
   - **(2)** Somewhat disagree
   - **(3)** Neither agree nor disagree
   - **(4)** Somewhat agree
   - **(5)** Strongly agree
   - **(99)** *I do not want to answer*
2. **PREP17d.** I would receive sexual health counseling every 3 months
   - **(1)** Strongly disagree
   - **(2)** Somewhat disagree
   - **(3)** Neither agree nor disagree
   - **(4)** Somewhat agree
   - **(5)** Strongly agree
   - **(99)** *I do not want to answer*
3. **PREP17e.** I would have a doctor check my health every 3 months
   - **(1)** Strongly disagree
   - **(2)** Somewhat disagree
   - **(3)** Neither agree nor disagree
   - **(4)** Somewhat agree
   - **(5)** Strongly agree
   - **(99)** *I do not want to answer*
4. **PREP17f.** I would have protection against HIV on a daily basis

- **(1)** Strongly disagree
- **(2)** Somewhat disagree
- **(3)** Neither agree nor disagree
- **(4)** Somewhat agree
- **(5)** Strongly agree
- **(99))** *I do not want to answer*

1. **PREP17g.** I would get the PrEP medication for free
   - **(1)** Strongly disagree
   - **(2)** Somewhat disagree
   - **(3)** Neither agree nor disagree
   - **(4)** Somewhat agree
   - **(5)** Strongly agree
   - **(99)9***I do not want to answer*
2. **PREP17k.** I would not have to rely on my partner using a condom to protect me against getting HIV

- **(1)** Strongly disagree
- **(2)** Somewhat disagree
- **(3)** Neither agree nor disagree
- **(4)** Somewhat agree
- **(5)** Strongly agree
- **(99)** *I do not want to answer*

1. **PREP17l.** The results of the study could help other teens

- **(1)** Strongly disagree
- **(2)** Somewhat disagree
- **(3)** Neither agree nor disagree
- **(4)** Somewhat agree
- **(5)** Strongly agree
- **(99)** *I do not want to answer*

1. **PREP17n.** I would be able to talk to research staff who are affirming of my sexual orientation

- **(1)** Strongly disagree
- **(2)** Somewhat disagree
- **(3)** Neither agree nor disagree
- **(4)** Somewhat agree
- **(5)** Strongly agree
- **(99)** *I do not want to answer*

**The following are reasons you might NOT want to participate in this type of study that tests whether PrEP pills or injections are the best way to protect teens who have sex with male partners from getting HIV. Please indicate how much you agree with these reasons.**

**I would NOT want to participate in this study because...**

1. **PREP18a** I do not want to know if I have HIV
   - **(1)** Strongly disagree
   - **(2)**  Somewhat disagree
   - **(3)** Neither agree nor disagree
   - **(4)** Somewhat agree
   - **(5)** Strongly agree
   - **(99)9***I do not want to answer*
2. **PREP18b** It would be too difficult to get to the appointments every few months
   - **(1)** Strongly disagree
   - **(2)**  Somewhat disagree
   - **(3)** Neither agree nor disagree
   - **(4)** Somewhat agree
   - **(5)** Strongly agree
   - **(99)9***I do not want to answer*
3. **PREP18d** If I was placed in the pill condition, I don't think I would remember to take the pills everyday
   - **(1)** Strongly disagree
   - **(2)**  Somewhat disagree
   - **(3)** Neither agree nor disagree
   - **(4)** Somewhat agree
   - **(5)** Strongly agree
   - **(99)9***I do not want to answer*
4. **PREP18g** I would feel the researchers were using me like a guinea pig
   - **(1)** Strongly disagree
   - **(2)**  Somewhat disagree
   - **(3)** Neither agree nor disagree
   - **(4)** Somewhat agree
   - **(5)** Strongly agree
   - **(99)** *I do not want to answer*
5. **PREP18h** I'm afraid other people would find out I was participating in the study
   - **(1)** Strongly disagree
   - **(2)**  Somewhat disagree
   - **(3)** Neither agree nor disagree
   - **(4)** Somewhat agree
   - **(5)** Strongly agree
   - **(99)** *I do not want to answer*
6. **PREP18i** I do not trust researchers to protect my confidentiality
   - **(1)** Strongly disagree
   - **(2)**  Somewhat disagree
   - **(3)** Neither agree nor disagree
   - **(4)** Somewhat agree
   - **(5)** Strongly agree
   - **(99)9***I do not want to answer*
7. **PREP18k** If I was placed in the injection condition, I wouldn't want to receive a shot
   - **(1)** Strongly disagree
   - **(2)**  Somewhat disagree
   - **(3)** Neither agree nor disagree
   - **(4)** Somewhat agree
   - **(5)** Strongly agree
   - **(99)** *I do not want to answer*

https://media.giphy.com/media/3o6Mb8gKNhqA59Fy3m/giphy.

1. **PREP19** Once the PrEP study ends, researchers cannot continue prescribing and providing PrEP, but they are able to provide a list of other doctors or clinics that can provide sexual health services, including HIV testing and PrEP, for LGBTQ teens. Knowing this, would you still want to participate in the PrEP study?
   - Definitely yes
   - Probably yes
   - Not sure
   - Probably no
   - Definitely no
   - **)** *I do not want to answer*
2. **PREP20** Now that you have read all about and how the PrEP study would work, would you want to participate in a PrEP study?
   - **(1)** Definitely yes
   - **(2)** Probably yes
   - **(3)** Not sure
   - **(4)** Probably no
   - **(5)** Definitely no
   - **(99)** *I do not want to answer*
3. **PREP21** Please tell us the most important reason for your decision to participate/not participate.

*If you do not wish to answer this question, type "no answer" in the text box.*

1. **PREP23** Even if I did not want to be in the study, I would agree to participate because it is the only way I can get PrEP for free.

- **(1)** Strongly disagree
- **(2)** Somewhat disagree
- **(3)** Neither agree nor disagree
- **(4)** Somewhat agree
- **(5)** Strongly agree
- **(99)** *I do not want to answer*

1. **ATTN03** To be sure you are still reading the content of these questions, please select "neither agree nor disagree" from the list of responses below.
   - **(1)** Strongly disagree
   - **(2)** Somewhat disagree
   - **(3)** Neither agree nor disagree
   - **(4)** Somewhat agree
   - **(5)** Strongly agree

]**We would like to get additional information about you so we can assess the characteristics of the teens who participate in our study. Please answer all questions as best as you can.**

1. **Demo15** What is your birthday?

. DROPDOWN DAY.

. DROPDOWN MONTH.

. DROPDOWN YEAR.

1. **Demo17** Are you currently a student?
   - **(1)** Yes, I'm in Middle School (6th-8th grade)
   - **(2)** Yes, I'm in High School (9th-12th grade)
   - **(3)** Yes, I'm in College
   - **(4)** No, I'm not in school
   - **(99)** *I do not want to answer*
2. **Demo18** Do you currently have a job?
   - **(1)** Yes, I work full-time
   - **(2)** Yes, I work part-time
   - **(3)** No
   - **(99)** *I do not want to answer*

**https://media.giphy.com/media/Wrv5v6egIh7Ww/giphy.gif**

**Thanks for your time!**
